# A Rare Case of Allergic Bronchopulmonary Aspergillosis With Endobronchial Aspergilloma Causing Total Lung Collapse

**DOI:** 10.7759/cureus.99810

**Published:** 2025-12-22

**Authors:** Shakithya Thavakumar, Mohamed A Abdulla

**Affiliations:** 1 Emergency Medicine, Chesterfield Royal Hospital, Chesterfield, GBR; 2 Respiratory Medicine, Chesterfield Royal Hospital, Chesterfield, GBR

**Keywords:** abpa, allergic bronchopulmonary aspergillosis, endobronchial aspergilloma, epa, total lung collapse

## Abstract

We present a rare case of allergic bronchopulmonary aspergillosis (ABPA) with endobronchial aspergilloma in a 70-year-old man who presented with acute total collapse of the left lung. Initial imaging raised concern for malignancy, but bronchoscopy revealed a fungal ball obstructing the left main bronchus. Serological markers confirmed ABPA. Treatment with bronchoscopy-guided clearance, oral corticosteroids, and voriconazole led to complete clinical and radiological recovery. This case highlights the importance of considering fungal aetiologies in atypical presentations of lung collapse.

## Introduction

Allergic bronchopulmonary aspergillosis (ABPA) is a hypersensitivity reaction to Aspergillus fumigatus, typically seen in patients with asthma or cystic fibrosis [1,2]. It is characterised by airway inflammation, eosinophilia, and elevated IgE levels, which can lead to bronchiectasis and lung damage if untreated [1,2]. Studies report that ABPA is found in approximately 2.5% of adults with asthma worldwide [3].

Aspergillomas usually form within lung cavities; however, endobronchial aspergilloma (EBA) is a rare manifestation in which fungal colonisation occurs within the bronchial lumen rather than the lung parenchyma [4-6]. EBA can present as an obstructive mass mimicking malignancy when associated with complete lung collapse [4-6]. Early recognition is crucial to avoid unnecessary oncological investigations and to initiate effective antifungal therapy [4,6].

## Case presentation

A 70-year-old gentleman presented to the emergency department with a one-day history of acutely worsening shortness of breath at rest, accompanied by a persistent cough. He denied chest pain, haemoptysis, or weight loss. Over the preceding month, he had experienced a three-week history of exertional dyspnoea, cough, and intermittent low-grade fever, which he self-managed with paracetamol. He also received a salbutamol inhaler, a course of antibiotics and steroids from his primary care doctor; however, his respiratory symptoms failed to improve and progressively deteriorated, prompting hospital admission.

His past medical history is notable for childhood asthma, though he has had no recent respiratory symptoms and no history of smoking. In 2006, he underwent a cystectomy for bladder cancer, followed by neobladder reconstruction using intestinal tissue.

He had occupational exposure to various chemicals and oils while working as an electrician and later worked in underground mining management. During the COVID-19 pandemic lockdown, he acquired an allotment and spent time working on it.

He had no exposure to farm animals or birds but did have some contact with wet grass on his allotment prior to the onset of his illness.

On examination, he was visibly breathless while speaking. There were absent chest movements and breath sounds on the left side, with dullness to percussion. Heart sounds were normal. Oxygen saturation was 88% on room air, requiring FiO₂ 50% to maintain the target oxygen saturation of >94%. Heart rate was 123 bpm, regular; blood pressure was elevated at 173/93. He was afebrile on admission. Abdominal and neurological examinations were unremarkable.

Initial investigations

The initial laboratory investigations are shown in Table 1.

**Table 1:** Initial laboratory investigations. eGFR: Estimated glomerular filtration rate

Parameter	Result	Reference Range
Haemoglobin	14.2 g/dL	13.5–17.5 g/dL
WBC	10.5 ×10⁹/L	4.0–11.0 ×10⁹/L
CRP	12 mg/L	<5 mg/L
Eosinophils	1.80 ×10⁹/L	0.0–0.5 ×10⁹/L
Platelets	250 ×10⁹/L	150–400 ×10⁹/L
Creatinine	65 mmol/L	59–104 mmol/L
eGFR	>90 ml/min/1.73m^2^	60-89 ml/min/1.73m^2^

Imaging

**Figure 1:**
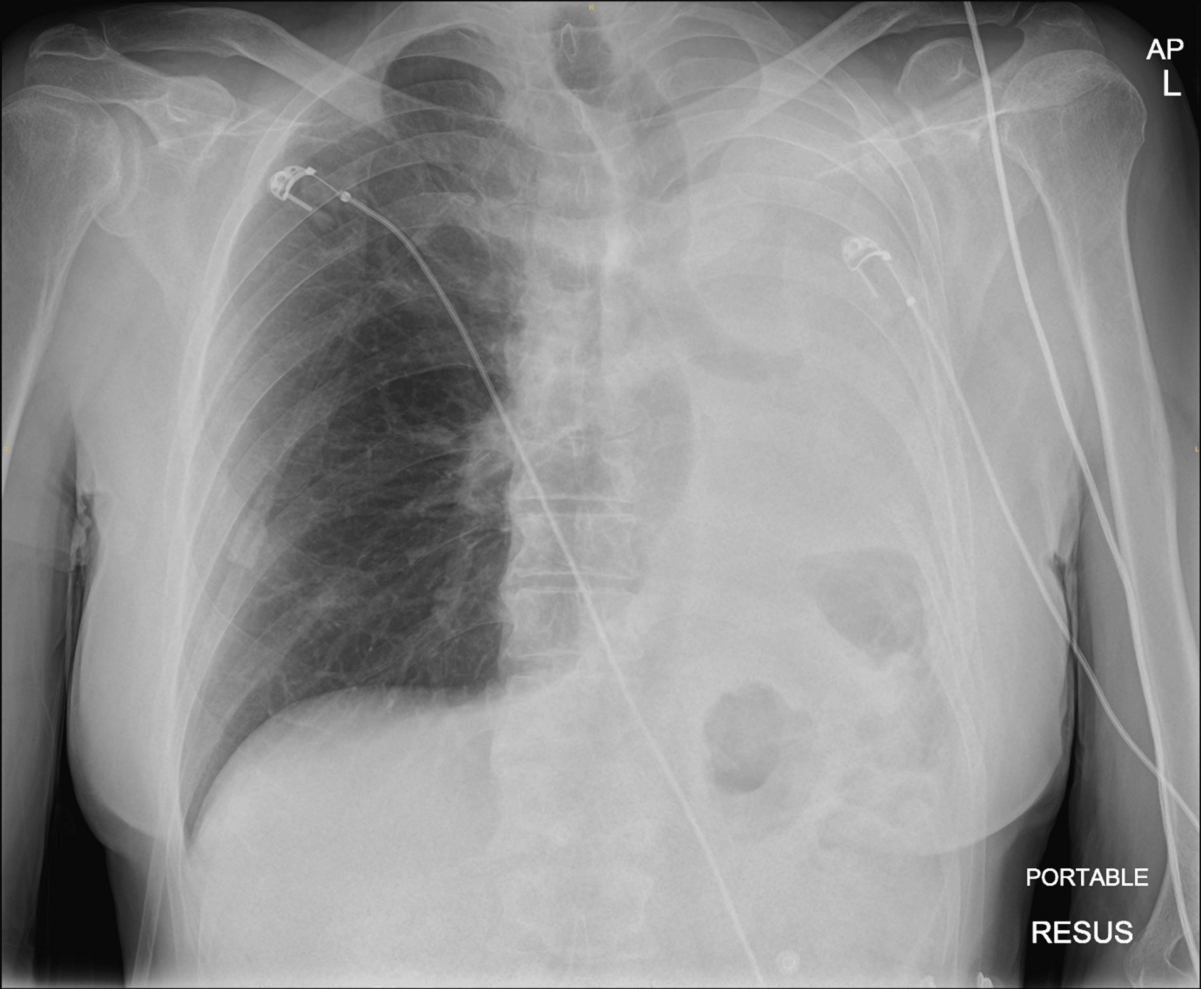
Chest X-ray showing complete white-out of the left lung with tracheal deviation.

Admission CXR showed complete white out of the left lung with tracheal deviation to the left indicating likely left lung collapse (Figure 1).

**Figure 2:**
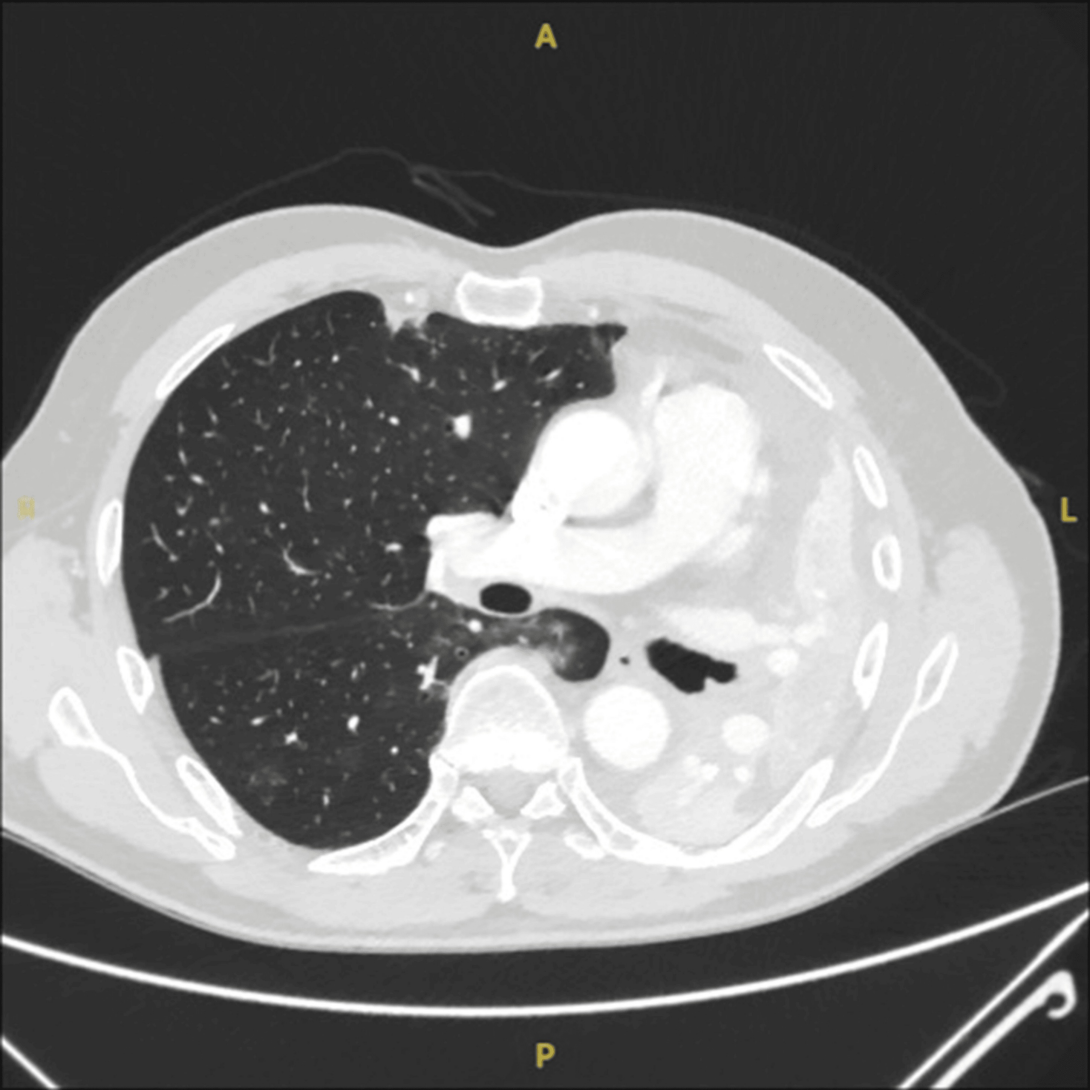
Slice of the CT scan just below the carina to show obstruction in the left main bronchus causing total left lung collapse.

**Figure 3:**
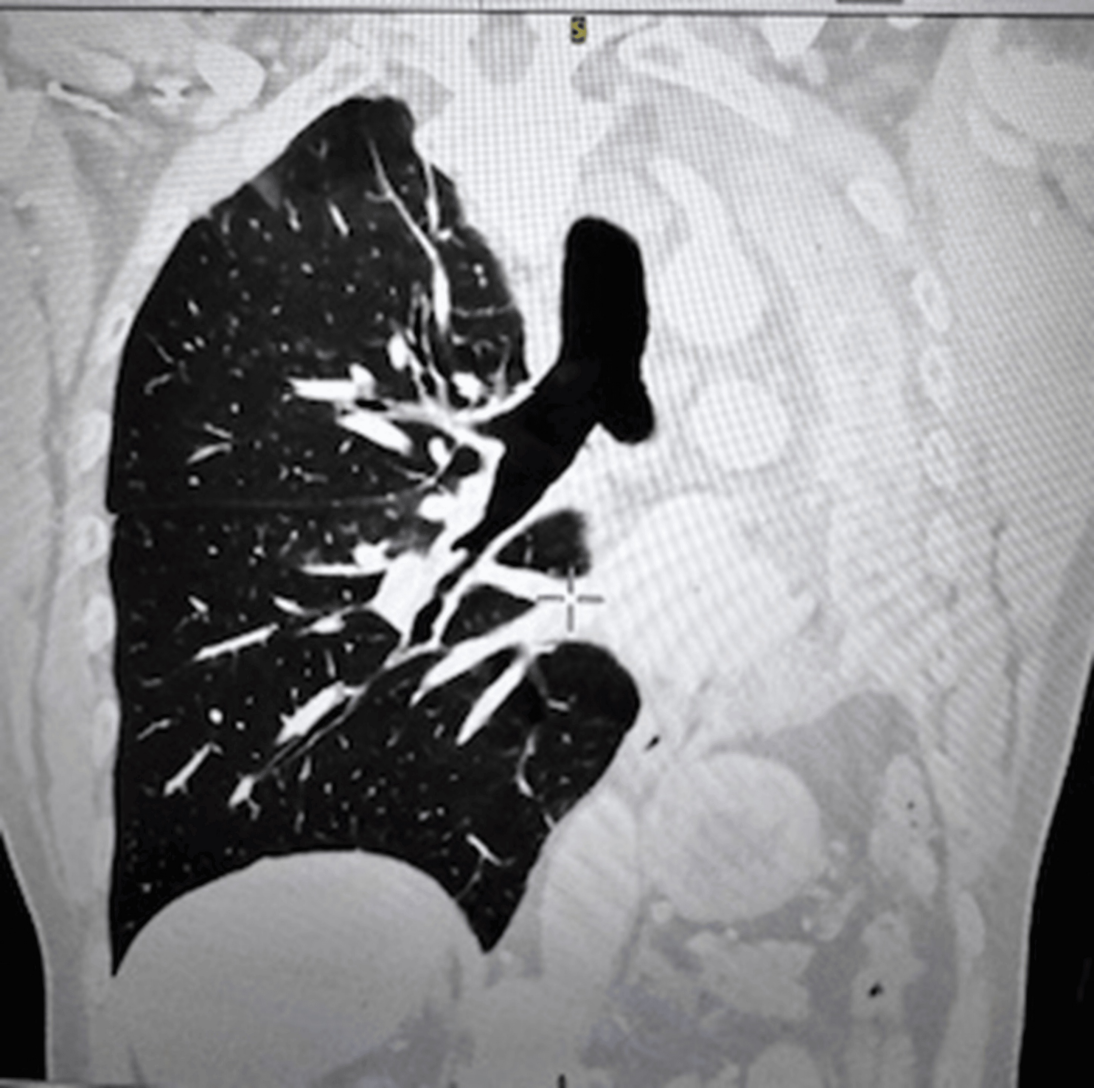
CT thorax in the coronal section showing complete obstruction in the left main bronchus and complete left lung collapse with hyperinflation of right lung and mediastinal shift to the left with left rib crowding.

CT Thorax/Abdomen/Pelvis confirmed complete collapse of the left lung with no mass identified (Figures 2, 3).

Initial diagnosis and management

Imaging raised suspicion for malignancy versus severe infection.

The patient was managed empirically with intravenous co-amoxiclav and oral clarithromycin, as well as supplemental oxygen to maintain adequate oxygenation.

Further investigations

The serology results are presented in Table 2.

**Table 2:** Serology results.

Test	Result	Reference Range
Total IgE	2855 IU/ml	<100 IU/ml
Aspergillus-specific IgE	3.69 kUA/L	<0.35 kUA/L
Aspergillus-specific IgG	107 mgA/L	<40 mgA/L
Vasculitis screen	Negative	Negative

The elevated total IgE and Aspergillus-specific IgE levels confirmed ABPA, while a negative vasculitis screen helped exclude other systemic inflammatory causes.

Due to the acute presentation with total lung collapse, urgent bronchoscopy was performed.

Bronchoscopy revealed a yellow, cheese-like fungal ball and mucus obstructing the left main bronchus. This provided both diagnostic confirmation and partial therapeutic clearance of the obstructing fungal mass. 

The bronchoalveolar lavage sample confirmed Aspergillus fumigatus growth in culture. Histology was unfortunately not obtained.

Treatment

Following the identification of Aspergillus fumigatus and elevated serological markers of ABPA, antifungal therapy was initiated with intravenous voriconazole, which was later transitioned to oral therapy. As per the British Thoracic Society guidelines, in patients with more rapidly progressive ABPA or those with a large volume of disease, the recommended first-line antifungal therapy is voriconazole 200 mg twice daily [7].

**Figure 4:**
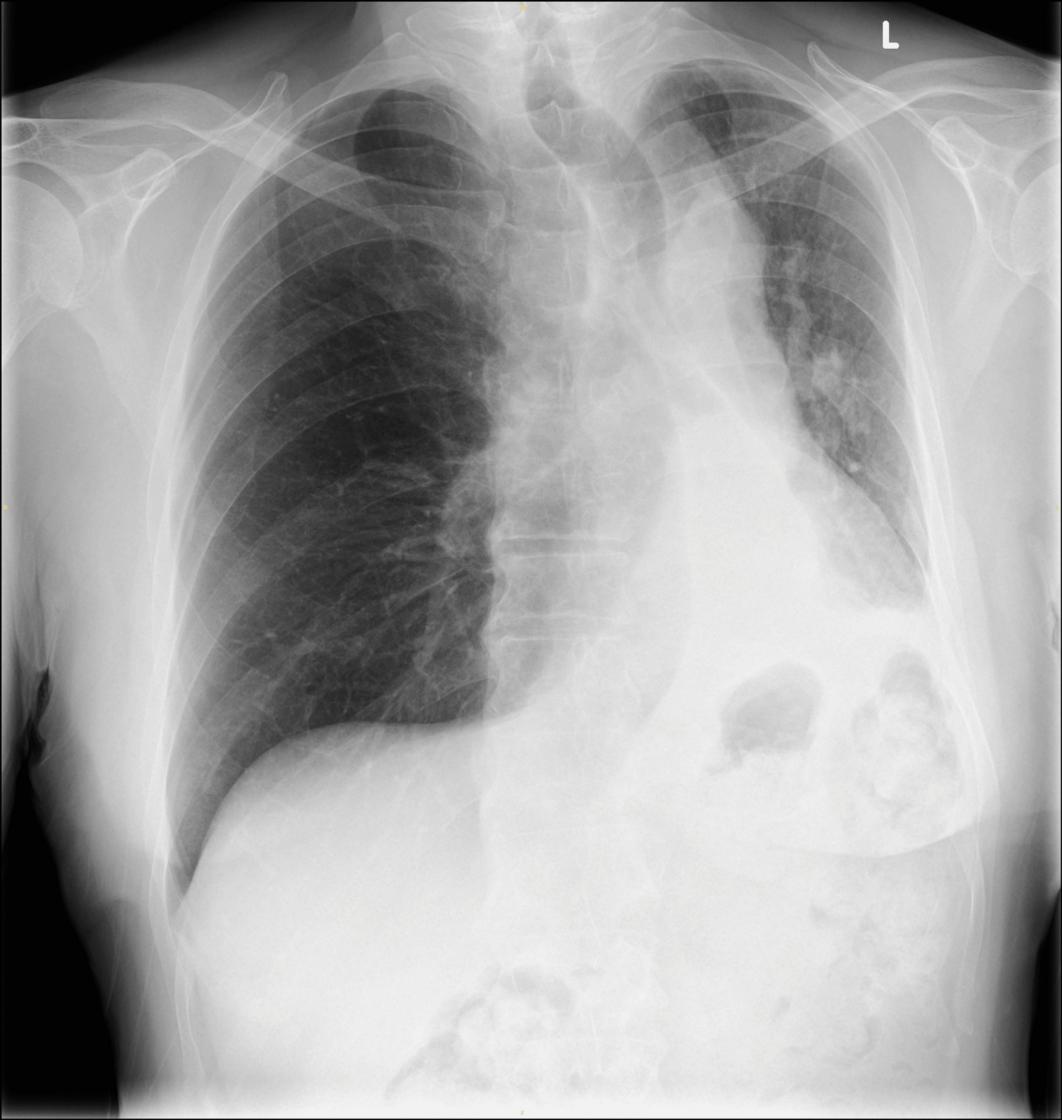
Chest X-ray performed on the day of discharge showing partial left lung re-expansion.

Oral corticosteroids (prednisolone 30 mg once daily) were started to manage the hypersensitivity component of ABPA. All antibiotics were discontinued once the fungal aetiology was confirmed. Oxygen supplementation was successfully weaned within 24 hours post-bronchoscopy, and post-procedure imaging showed partial re-expansion of the left lung (Figure 4).

Outcome and follow-up

The patient was discharged on oral voriconazole for three months and a tapering corticosteroid regimen, with regular outpatient monitoring of liver function and serum drug levels by the respiratory pharmacist.

**Figure 5:**
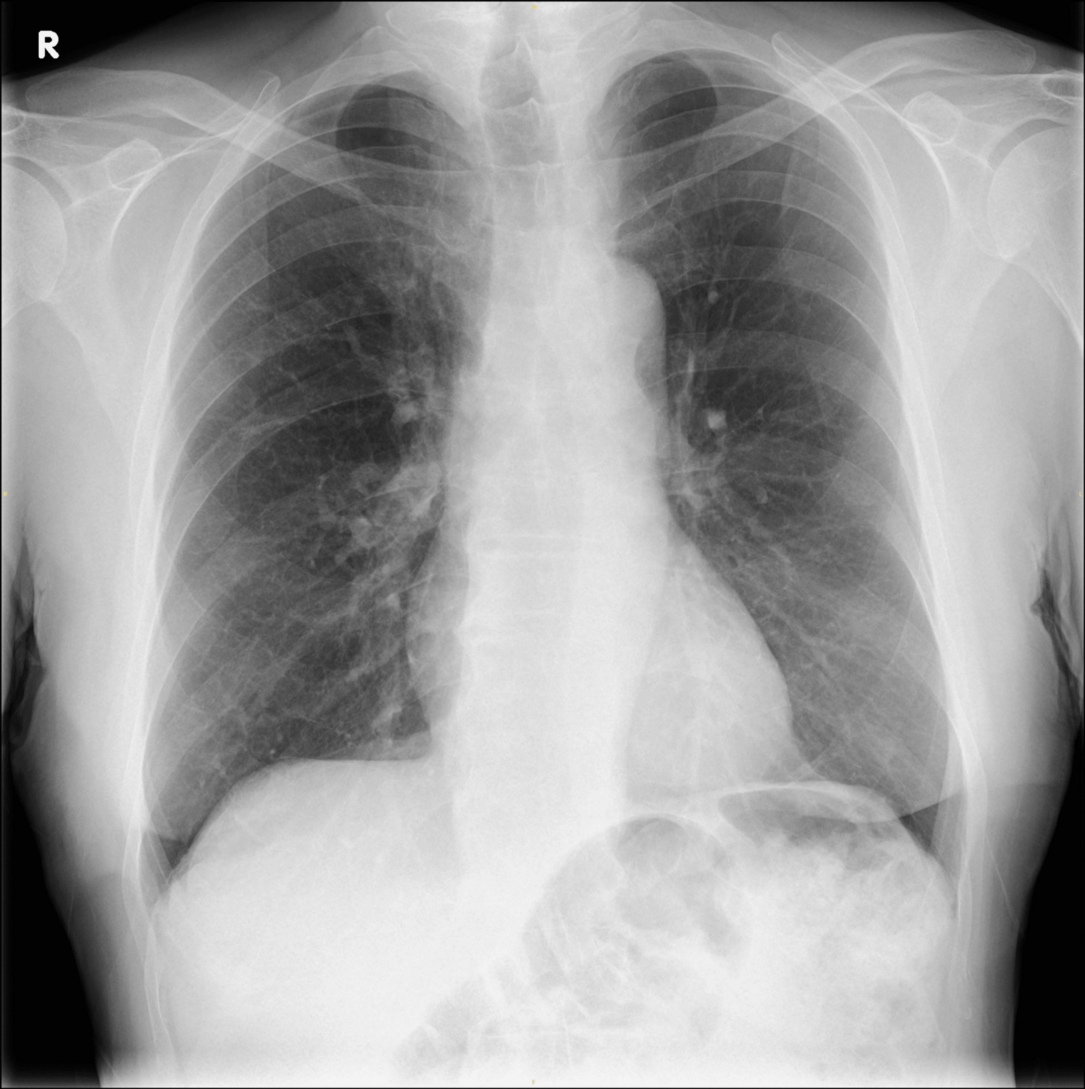
Chest X-ray performed at the three-month follow-up in the respiratory clinic showing complete re-inflation of the left lung.

 At follow-up, he had complete symptom resolution, normalised eosinophil count with normal chest X-ray (Figure 5) and successful steroid weaning.

## Discussion

EBA is a rare form of pulmonary aspergillosis, characterised by fungal growth within the bronchial lumen rather than the parenchyma [4-6]. Unlike classical aspergillomas, which typically form within pre-existing lung cavities, EBA produces endobronchial obstruction composed of fungal hyphae, mucus, and necrotic debris. This form of disease is often misdiagnosed due to its radiological resemblance to malignancy, especially when presenting with complete lung collapse [4,6,8].

Most reported cases occur in individuals with underlying lung conditions, including previous tuberculosis, bronchiectasis, or immunosuppression [5,8]. Our patient had no active asthma or structural lung disease, making this presentation particularly unusual. The absence of haemoptysis, weight loss, or systemic symptoms further complicated the initial diagnosis, leading to a working differential of malignancy versus infection.

Bronchoscopy plays a pivotal role in both diagnosis by allowing direct visualisation and management for relieving the obstructing fungal mass [1,4,9]. In our case, the visual identification of a cheesy yellow mass in the left main bronchus, along with positive cultures for Aspergillus fumigatus, confirmed the diagnosis. Histology was not obtained, which is a limitation, but serological markers, including elevated total IgE and Aspergillus-specific IgE, supported ABPA with EBA [2].

EBA is more frequently found in the left lung, possibly due to anatomical predisposition related to bronchial angulation and airflow patterns [10].

Reviews have shown that a combined management of bronchoscopy intervention and antifungal therapy, most commonly voriconazole, was effective in achieving resolution [1,5,6,9]. Corticosteroids are indicated in ABPA to control immune-mediated inflammation and prevent disease progression [2,6].

Another comprehensive review emphasised the importance of early bronchoscopy and fungal culture to avoid misdiagnosis and unnecessary oncological investigations [2].

## Conclusions

This case highlights the importance of considering fungal aetiologies, including EBA, in patients presenting with acute lung collapse, even in the absence of classical risk factors.

Early bronchoscopy and fungal serology are essential for accurate diagnosis. Combined management with bronchoscopic clearance, systemic antifungal therapy, and corticosteroids can lead to complete clinical and radiological recovery, emphasising that EBA is a rare but treatable cause of airway obstruction.
